# De novo assembly of the complex genome of *Nippostrongylus brasiliensis* using MinION long reads

**DOI:** 10.1186/s12915-017-0473-4

**Published:** 2018-01-11

**Authors:** David Eccles, Jodie Chandler, Mali Camberis, Bernard Henrissat, Sergey Koren, Graham Le Gros, Jonathan J. Ewbank

**Affiliations:** 1 Malaghan Institute of Medical Research, Wellington, New Zealand; 2 Department of Biological Sciences, King Abdulaziz University, Jeddah, Saudi Arabia; 3 CNRS UMR 7257, Aix-Marseille University, Marseille, France; 4 INRA, USC 1408 AFMB, Marseille, France; 5 Genome Informatics Section, Computational and Statistical Genomics Branch, National Human Genome Research Institute, National Institutes of Health, Bethesda, MD 20892 USA; 6 Centre d’Immunologie de Marseille-Luminy, Aix-Marseille University, CNRS, INSERM, Marseille, France

**Keywords:** Helminths, Next-generation sequencing, Base-calling, Genome assembly, DNA repeat, Population analysis

## Abstract

**Background:**

Eukaryotic genome assembly remains a challenge in part due to the prevalence of complex DNA repeats. This is a particularly acute problem for holocentric nematodes because of the large number of satellite DNA sequences found throughout their genomes. These have been recalcitrant to most genome sequencing methods. At the same time, many nematodes are parasites and some represent a serious threat to human health. There is a pressing need for better molecular characterization of animal and plant parasitic nematodes. The advent of long-read DNA sequencing methods offers the promise of resolving complex genomes.

**Results:**

Using *Nippostrongylus brasiliensis* as a test case, applying improved base-calling algorithms and assembly methods, we demonstrate the feasibility of de novo genome assembly matching current community standards using only MinION long reads. In doing so, we uncovered an unexpected diversity of very long and complex DNA sequences repeated throughout the *N. brasiliensis* genome, including massive tandem repeats of tRNA genes.

**Conclusion:**

Base-calling and assembly methods have improved sufficiently that de novo genome assembly of large complex genomes is possible using only long reads. The method has the added advantage of preserving haplotypic variants and so has the potential to be used in population analyses.

**Electronic supplementary material:**

The online version of this article (10.1186/s12915-017-0473-4) contains supplementary material, which is available to authorized users.

## Background

Human hookworm infections by the parasitic nematodes *Necator americanus* and *Ancylostoma duodenale* continue to be a major global health problem. Next-generation sequencing (NGS) techniques open the door to molecular epidemiological monitoring of nematode and helminth parasites in endemic areas. Such studies are, however, hampered by the heterogeneous nature of parasite populations and by the intrinsically complex genome structures of nematodes [1]. In contrast to most NGS machines, which are cumbersome and can be operated only within a laboratory setting, Oxford Nanopore Technology (ONT) MinION sequencers are small and highly portable. They are robust and are now being used all over the globe, even in extreme environments [2, 3]. That they are well suited for field studies, and their capacity to generate long-read sequences, makes them potentially an ideal tool for conducting molecular epidemiological studies of nematode and helminth parasites in remote locations.

Long-read sequences are especially useful for de novo genome assembly. Reads from the MinION, as well as from PacBio’s single-molecule real-time (SMRT) sequencing platform, however, suffer from an error rate that is very substantially higher than seen with short-read NGS technologies. As a consequence, de novo genome assembly based on long DNA reads often relies on hybrid strategies incorporating, for example, short-read DNA sequencing [4–7], although non-hybrid long-read only methods do exist [8–12] (see [13] for a review). Indeed, there have now been successful chromosome-scale assemblies of large genomes, primarily using the PacBio SMRT platform to generate contigs (e.g. [14]), combined with long-range linking information (e.g. [15, 16]) but the approach can still be challenging.

**Fig. 1:**
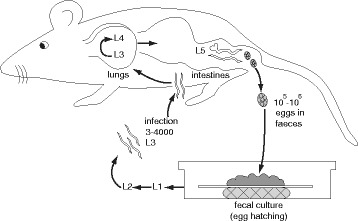
Life cycle of *Nippostrongylus brasiliensis* in the laboratory. Parasite stages are not drawn to scale. Adapted with permission from Camberis et al. [35]

As a model for the analysis of human hookworm populations, we turned to *Nippostrongylus brasiliensis*, a gastrointestinal nematode that infects rodents. Its lifecycle is analogous to that of hookworms and it is widely used as a surrogate for hookworms in research (e.g. [17]). Lines of *N. brasiliensis* are maintained by serial passage in rats. The standard culture protocol involves infection of several rats by thousands of infective larvae. Each rat will produce hundreds of thousands of eggs in a few days [18]. These are harvested in feces and grown without any intentional selection to give a new generation of infective larvae (Fig. 1). Although there will be some adaptation of this laboratory-maintained strain to rats housed in a specific pathogen-free animal facility, the use of this relatively high number of worms at each generation is expected to maintain diversity within the population. We, therefore, took *N. brasiliensis* as a test case to evaluate the possibility of generating a genome sequence de novo from a heterogeneous population using MinION long reads and improved analysis methods.

## Results and discussion

In the context of the 50 Helminth Genomes Initiative [19], the Wellcome Trust Sanger Institute (WTSI) assembled a reference genome for *N. brasiliensis* using Illumina paired-end short-read sequencing (parasite.wormbase.org/Nippostrongylus_brasiliensis_prjeb511). This is a highly fragmented sequence (Table 1); almost 30% of predicted protein-coding genes (6276/22,796) are on contigs that are less than 10 kb long. To estimate the size of the *N. brasiliensis* genome, we fed the WTSI Illumina reads into GenomeScope [20]. Although its algorithms are designed for heterozygous genomes, not sequences from a genetically diverse population, it can give a rough guide to genome size in such cases. GenomeScope tends to underestimate genome size. For example, it returns a size for the *Drosophila melanogaster* genome of 130 Mb, about 75–80% of the typical long-read assembly (SK, unpublished). With the WTSI Illumina reads, GenomeScope returned 205 Mb for the total length of unique genomic sequences. We, therefore, predicted a genome of approximately 255–270 Mb for *N. brasiliensis*.

**Table 1:** Genome and transcriptome contiguity scores. Statistics for the current WTSI reference sequence are given for comparison

Description	Size (kb)	N50 (kb)	N90 (kb)	Minimum (kb)	Maximum (kb)
	[Contigs/genes]	[L50]	[L90]		
WTSI reference	294,400	33.5	4.3	0.5	394.2
	[29,375]	[2024]	[11,638]		
Uncorrected (Canu only)*	347,186	209.2	38.9	1.7	2048.3
	[3583]	[415]	[1964]		
Trinity [de novo]	180,448	0.8	0.3	0.2	21.5
	[291,671/172,480]**	[53,613]	[219,557]		
Trinity [genome-guided]	249,065	1.2	0.3	0.2	22.0
	[352,994/236,865]**	[50569]	[245,765]		
Trinity [expression-filtered]	71,457	2.0	0.7	0.2	22.0
	[52,302]	[11,072]	[34,924]		
WGA, Canu only	119,196	53.1	17.3	1.8	388.2
	[3280]	[647]	[2199]		

*WGA* whole-genome amplification, *WTSI* Wellcome Trust Sanger Institute

*Local read correction has minimal effect on the contiguity statistics

**In the absence of expert curation, these values are patently overestimates (e.g., in the genome-guided assembly, genes near structural variant forks will be counted more than once)

**Fig. 2:**
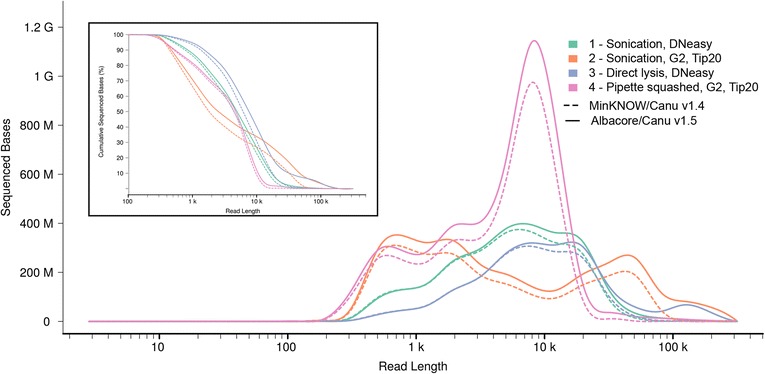
Effect of extraction and analysis methods on the size of DNA reads. The distribution of read lengths for unamplified DNA extracted by each one of four methods and using two analysis methods is shown. The insert is a cumulative plot of the same data

We sequenced DNA extracted from a population of adult *N. brasiliensis* resident in the small intestine of a rat using R9.4 flow cells on a MinION Mk1b. DNA preparation and sequencing was managed by ONT. Four sample preparation methods were used (see ‘Methods’ for details), which gave very different results both in terms of yield, which varied more than twofold in total, up to 4.7 Gb for a single run, and in read length distribution (Fig. 2, Table 2 and Additional file 1: Table S1). The original base-calling using MinKNOW (carried out in January 2017) produced 4,911,193 reads totaling 10.2 Gb of sequence. We then recalled the same raw nanopore data using the more recent Albacore (v1.1.0), a production base caller that implemented a transducer algorithm for homopolymer detection, previously a persistent limitation in the analysis of MinION reads [21]. Overall, we obtained 5,472,882 called reads for a total of 12.3 Gb of sequence. This represents a >20% increase in yield over the previous approach. In all cases, Albacore outperformed the older base caller, although, interestingly, the improvement was far from uniform, varying from less than 10% to greater than 30% in the total length of reads called for the different samples. There was also a noticeable increase in the proportion of very long (>50 kb) reads, in one case reaching close to 0.4% of all reads, and with a more than 50-fold increase in reads over 100 kb (Fig. 2, Table 2 and Additional file 1: Table S1). This is potentially of great utility for genome assembly. Similar observations have been made in a very recent comparison of different base-callers (https://doi.org/10.5281/zenodo.1043611).

**Table 2:** Yields of sequences using different extraction and analysis methods. The percentage of the raw reads that were included in the output of the two methods (MinKNOW and Canu v1.4, or Albacore and Canu v1.5) is shown together with the total sequence lengths and the number of reads exceeding the indicated lengths. See ‘Methods’ for experimental details and Additional file 1: Table S1 for more information

	Yield								
Preparation Method	Raw reads	MinKNOW				Albacore			
		%	Gb	>50 kb	>100 kb	%	Gb	>50 kb	>100 kb
1. Sonication, DNeasy	930,957	99.9	2.375	80	15	99.1	2.603	472	82
2. Sonication, G2, Tip20	1,845,520	85.2	2.289	2252	17	98.0	3.002	4888	1006
3. Direct lysis, DNeasy	451,578	99.9	1.723	179	26	98.8	2.028	1710	775
4. Pipette squashed, G2, Tip20	2,333,064	83.9	3.834	10	0	98.4	4.678	427	89

**Fig. 3:**
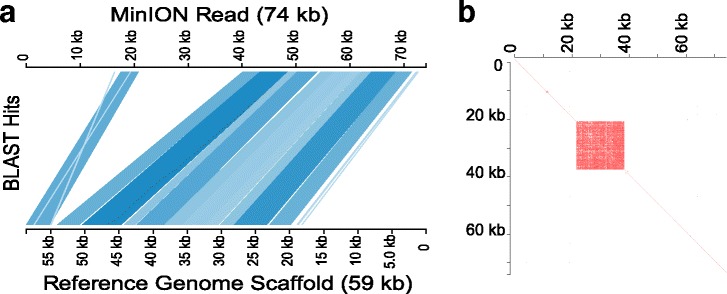
Improved sequence fidelity using the updated base caller. **a** Alignment of a single 74-kb read against the corresponding scaffold of the current WTSI reference genome, plotted using Kablammo [36]. **b** Identification of a very long stretch of complex tandem repeats (21 kb with a 171-bp repeat unit) within the same read. WTSI Wellcome Trust Sanger Institute

As a gauge of the accuracy of Albacore read-calling, we compared a random subset of the longer reads with the WTSI *N. brasiliensis* reference genome. While overall there was generally a good equivalence, especially given the potential genetic difference between the two samples (see ‘Methods’), there were numerous instances where reads were substantially longer than the matching sequence in the WTSI genome. Upon further examination, some of these were revealed to reflect the presence of very long stretches of complex tandem repeats (VeCTRs) that had been compacted in the WTSI genome (Fig. 3).

**Fig. 4:**
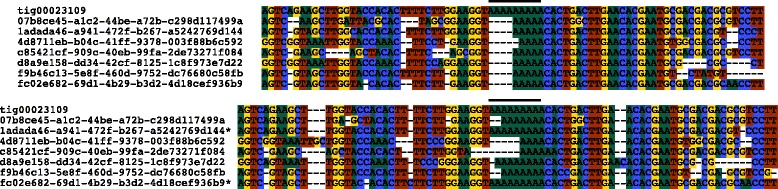
Improved sequence fidelity using updated methods. Homopolymeric regions that were compressed in the original MinKNOW-called sequence (top) were expanded when the base caller Albacore included a transducer mode that incorporates signal length into the called sequence (bottom). The consensus sequence from the final assembly (from contig tig00023109) is shown together with the same set of individual reads, called with MinKNOW (top) or Albacore I (bottom). Sanger sequencing of PCR amplicons confirmed the accuracy of the newer base-calling, specifically the stretch of nine A’s, marked by the black bar, which was not supported by a single read in the MinKNOW data. Some of the sequence variation here may reflect genuine genotypic variation and not simply sequencing errors

**Fig. 5:**
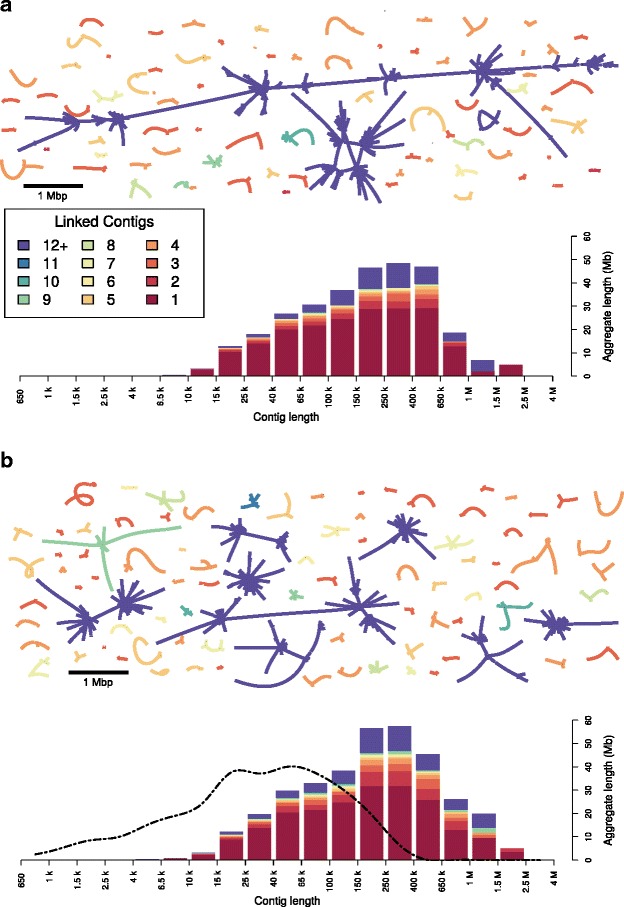
Improved assembly using updated methods. Distribution of fragment sizes and complexity for contigs assembled using old (MinKNOW/Canu v1.4; **a**) and new (Albacore/Canu v1.5; **b**) methods with unamplified DNA. The structures of the assembled contigs (drawn to scale, as indicated) are also shown. The unbranched subgraphs representing more than 70% of the sequence (dark magenta bars) have been omitted. The distribution of fragment sizes from the WTSI reference genome (dashed dotted line) is shown in (**b**). The largest two subgraphs in (**a**) contain 207 contigs (total of 28.7 Mb) and 101 contigs (17.3 Mb) and in (**b**) 106 contigs (13.3 Mb) and 76 contigs (10.6 Mb). In addition to the better resolution of subgraphs, in the new assembly, there is a marked increase in unbranched subgraphs longer than 1 Mb. This improvement in contiguity reflects the increase in the quality and quantity of called reads, and better assembly algorithms. WTSI Wellcome Trust Sanger Institute

We combined the Albacore-called reads from the four samples and fed them into Canu v1.5, an update of the assembler Canu [22], which has improved read trimming, read correction, and consensus calling, and provides more accurate graph information in the assembly output. We noted local improvements in the calling of homopolymer sequences compared to the MinKNOW-derived sequences (Fig. 4). We obtained an assembly of close to 350 Mb, with a maximum contig length of >2 Mb and an N50 of 209 kb (Table 1). A total of 251.6 Mb (72.5%) of the sequence, including all of the longest (>1.5 Mb) contigs, was contained within 2594 non-branched subgraphs of the Canu assembly graph, with 2413 of them made from a single contig, and the others containing an average of 2.15 linearly-linked contigs. The remaining 95.6 Mb was captured by a total of 780 contigs in 89 branched subgraphs. Overall, there were marked improvements in the quality of the assembly, compared to what was generated using the MinKNOW-called sequence and Canu v1.4, in terms of contig length and graph complexity (Fig. 5). A small number of the 89 branched subgraphs obtained with Canu v1.5 still had very complex structures (Fig. 5b). Inspection showed this to result from the presence of long non-tandem repeat sequences. Indeed this, together with real sequence diversity (see below), was the most common cause of assembly ambiguity. Resolving such structures would require a greater depth of coverage and/or even longer reads, and would be greatly simplified if the DNA from individual animals could be sequenced (see below).

**Fig. 6:**
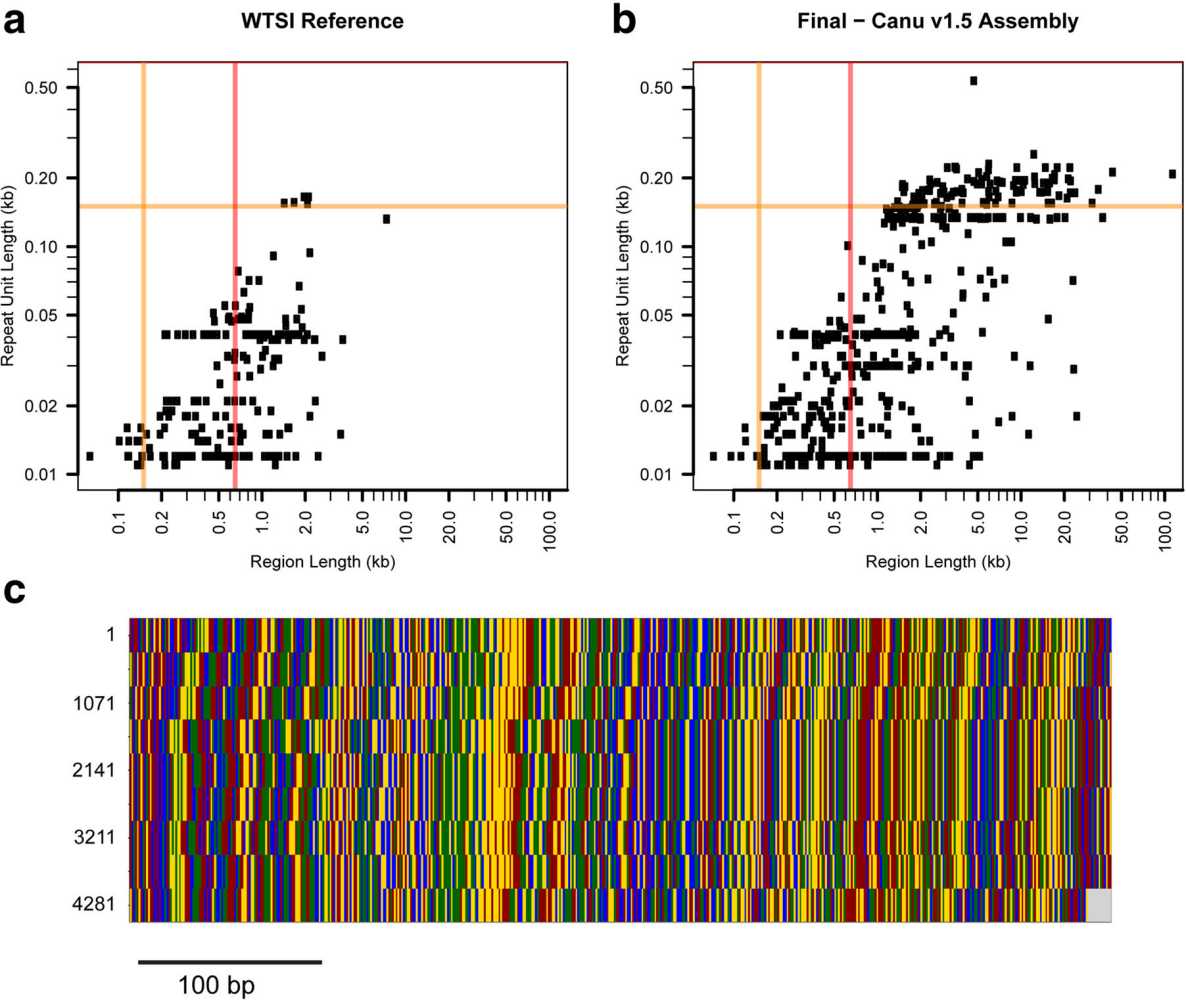
Analysis of repeat sequences within different assemblies. SATFIND [23] was used on contigs >2.5 kb. The total length of each region of a repeated DNA sequence is plotted against the repeat’s unit length, for the WTSI genome assembly (**a**) and our final assembly (**b**). The orange and red lines are at 150 bp (typical maximum read length for an Illumina HiSeq run) and 650 bp, respectively. Any VeCTRs with a unit length longer than 150 bp would not be identifiable as a repetitive sequence on an Illumina sequencer. Any VeCTRs with a region length longer than the maximum target fragment length for Illumina sequencing (typically 650 bp) will be collapsed into a shorter region if only non-mate-paired 150 bp reads are used for the assembly. **c** Alignment of the region corresponding to the longest repeat unit length in (**b**), with each base represented as a colored line. Although the resolution of repeats is greatly improved compared to the WTSI assembly, such sequences still present a challenge for assembly, as evidenced by the fact that the 5' end of this sequence corresponds to a contig end (tig00023164; coordinates on the left). The color code is as in Fig. 4. Very long stretch of a complex tandem repeat (VeCTR), Wellcome Trust Sanger Institute (WTSI)

The final assembly contained a much greater diversity of repeat sequences than seen in the WTSI reference sequence (Fig. 6a,b). The repeat with the longest unit length (535 bp) as determined by Satfind [23], corresponded to a region with ten tandem copies (Fig. 6c) of an 5S rRNA gene interspersed with an snRNA gene, the source of the spliced leader RNA that is added to many transcripts. The gene sequences and this arrangement are conserved well in *Caenorhabditis elegans*, where these pairs are repeated over a region of 16 kb (V:17,118,000..17,132,000; WS260). In the WTSI assembly, on the other hand, equivalent sequences, in single copy, were found at contig ends, reflecting the difficulty they pose for short-read-based assembly. There were also transposon-associated repeats, the repeats that correspond to reiterated amino acid motifs in proteins, and the dispersed satellite DNA sequences found in holocentric nematodes [23]. For the other VeCTRs, their length and the constituent repeated sequences were diverse. There was, for example, no sequence similarity among the five most compressible VeCTRs (Additional file 2: Figure S1). The longest of them comprised close to 150 copies of an approximately 200-bp repeat, with the (conserved) tRNA-Trp gene, followed by a sequence currently unique to *N. brasiliensis*. Similarly, the shortest of the five corresponded to 90 copies of the tRNA-Ser gene interspersed with a *N. brasiliensis*-specific 80-bp spacer. In *C. elegans*, there are more than 600 tRNA genes. Some are clustered in small groups but never with such a massively repeated organization. The other three VeCTRs contain a sequence that is not conserved. *C. elegans* genomic DNA has also been sequenced directly using long-read techniques [24]. Remarkably, VeCTRs have now also been found in the *C. elegans* genome, where they account for more than 1 Mb of sequence omitted from the current N2 reference genome (using VC2010, a strain derived from a clonal isolate of N2; E. Schwarz, personal communication), which was assembled primarily by Sanger sequencing of inserts from cosmid libraries [25]. Since they are not amenable to either short-read NGS or traditional cloning, they may have been overlooked in other species and potentially have specific but as yet unknown biological roles.

**Fig. 7:**
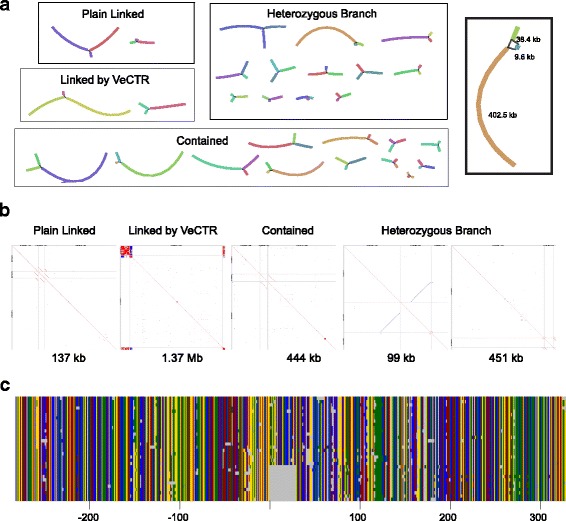
Classification of subgraphs and identification of a haplotype signature. **a** Bandage plots for the 28 simple GFA subgraphs made of three contigs. The box on the right is an enlarged view of a heterozygous branch subgraph with a total length of 451 kb. **b** Dot plots of LAST all-against-all minimum-distance sequence comparisons between the three constituent contigs, delineated by the gray lines, for representatives for each of the subgraph classes. The sum of the contig lengths is indicated. The 451-kb subgraph is that enlarged in (**a**). **c** Multiple alignment of contig sequences from this subgraph and corresponding region of the DNA reads that map to them, 300 bp either side of the defining deletion. The color code is as in Fig. 4. Graphical Fragment Assembly (GFA) (file format); describes the assembly graph, Very long stretch of a complex tandem repeat (VeCTR)

Returning to the analysis of the branched subgraphs, among the less complex ones, 28 linked just three contigs. Of these, two were due to the presence of VeCTRs and in two, one contig matched the extremities of two non-overlapping contigs, but in a redundant manner (Plain Linked) (Fig. 7a,b). The others showed structures compatible with multiple haplotypes within the helminth population. Of these, 12 had two contigs with homologous end sequences converging on a common contig (contained), a configuration that can also arise when base-calling errors are high. The remaining 12 had stronger support for being genuine haplotypes, including three subgraphs with a bubble structure indicative of haplotype resolution, as supported by examination of the underlying reads (Fig. 7). While attempting to identify a primary genomic haplotype might have some use in determining the true contiguity of the assembly and estimating the real genome size, this assembly represents a community sample and preserves the observed read variation.

In addition to this ability to uncover haplotypes within a population, overall, there was an impressive increase in the quality of the genome assembly compared to the current WTSI genome by standard contiguity criteria (Fig. 5 and Table 1), although the WTSI genome scored substantially higher when evaluated by Benchmarking Universal Single-Copy Orthologs (BUSCO) [26] (Table 3). On the other hand, BLAST searches of our assembly for the missing universal single-copy ortholog (USCO) genes identified plausible orthologs in most cases (see below). This suggested that even if assembly continuity were improved, the local sequence quality would not be sufficiently high to allow correct gene prediction and ortholog identification by BUSCO. Nanopolish can improve the consensus sequence for draft genome assemblies, using just raw MinION reads [27]. Applying Nanopolish to our assembly resulted in a clear increase in sequence quality, as judged by the marked rise in the predicted USCO genes (from 65% to 85% complete), exceeding even the WTSI value (80.1%; Table 3).

**Table 3:** BUSCO scores following different methods of genome sequence correction. The percentage of complete USCOs together with the number of single, duplicated, fragmented (Frag) or missing USCO predicted from the uncorrected genome sequence, or the sequence after processing by different methods (see ‘Methods’ for details). The figures for the WTSI reference are shown for comparison. The default analysis mode is the short one. In the long mode, an optimization of the BUSCO sub-program Augustus is used for self-training, substantially increasing run times

	Short BUSCO					Long BUSCO				
Description	Complete (%)	Single (*n*)	Duplicated (*n*)	Fragmented (*n*)	Missing (*n*)	Complete (%)	Single (*n*)	Duplicated (*n*)	Fragmented (*n*)	Missing (*n*)
Uncorrected (Canu only)	49.2	437	46	128	371	65.0	585	53	122	222
Nanopolish						85.4	733	106	73	70
Bowtie2 + Pilon	75.6	630	112	95	145	85.1	709	127	72	74
HISAT2 + Pilon	74.7	618	116	101	147	81.5	679	122	83	98
Trinity^#^ [de novo]	*90.8* 90.7	*681* 441	*211* 450	*65* 64	*25* 27	91.3	446	451	59	26
Trinity^#^ [genome-guided]	*88.4* 88.2	*316* 304	*552* 562	*63* 61	*51* 55	88.5	305	564	58	55
Trinity^#^ [Expression-filtered]	*87.9* 87.7	*644* 613	*219* 248	*58* 60	*61* 61	88.3	618	249	55	60
Trinity^#^ [collapsed]	*87.2* 87	*786* 791	*71* 64	*58* 63	*67* 64	87.6	796	64	57	65
WTSI reference	73.1	677	41	133	131	80.6	748	43	125	66

*USCO* universal single-copy ortholog (genes), *WTSI* Wellcome Trust Sanger Institute

^#^Statistics in italics are from transcript mode BUSCO

Many recent studies have used short-read NGS DNA or cDNA data for local genome sequence correction. We used a set of Illumina RNA-seq reads generated from polyA cDNAs from our *N. brasiliensis* strain (GLG et al., unpublished) to correct the sequence of protein-coding genes, while not touching the genome assembly. Pilon [28] in combination with Bowtie2-based alignment, using the set of genome-guided Trinity transcript sequences, gave the best results as judged by the markedly improved BUSCO scores (with 88% complete), close to the score of 91% complete USCO genes for the de novo assembly from the same RNA-seq reads (Table 3). Our results indicate that the RNA-seq reads cover a substantial fraction of the transcriptome, and that the genome also has excellent coverage of most of the expressed *N. brasiliensis* genes. Within the assembled consensus sequence corresponding to expressed transcripts, about 1% of bases were locally corrected using the cDNA reads. The most frequent consensus errors correctable by Pilon were single-base deletions (44%), which were tenfold more frequent than single-base insertions, while single-base transitions (A–G or C–T) accounted for 28% of the corrections (Table 4). Interestingly, although there was an overlap for a core group of 34 missing USCO genes from the uncorrected, Nanopolish-, and Trinity [genome-guided]-corrected assemblies, the methods gave very complementary results (Additional file 3: Figure S2A). Even among the core group, BLAST searches revealed putative orthologs for most of the missing genes. For six of them, the BLAST alignments were associated with *E* scores of <1 × 10^-15^ (Additional file 3: Figure S2b–e; Additional file 4: Table S2). These results, thus, confirm that single-base errors, generated using the combination of Albacore and Canu, were the principal cause of the lower BUSCO scores seen for the unpolished genome assembly. In many studies, short-read sequences will not be available to guide local genome sequence correction. Our results also show that the sequence obtained using only long reads comes close to that obtained with hybrid strategies. Unlike short-read-based strategies, a method like Nanopolish can contribute to the full description of genome complexity. It is capable of resolving the structures of near-identical genes present in multiple genomic regions and can also improve the consensus sequence in repeat regions. The continuing progress in base-calling and polishing methods promises to make short-read-based correction superfluous (see also https://doi.org/10.5281/zenodo.1043611).

**Table 4:** Sequence correction statistics. The number and proportion of classes of sequence corrections are given, calculated using the total number of genome bases mapped by the cDNA reads at coverage >10 (77,335,202). INDELs of three or more were binned together as their individual contribution to variance was less than 1%

Count	Proportion (of variants)	Proportion (of bases)	Original	Corrected
312,140	44.2%	0.404%	.	N [1 bp INS]
65,725	9.3%	0.085%	A	G
64,702	9.2%	0.084%	T	C
48,786	6.9%	0.063%	..	NN [2 bp INS]
33,471	4.7%	0.043%	C	T
33,348	4.7%	0.043%	G	A
33,163	4.7%	0.043%	N	. [1 bp DEL]
19,452	2.8%	0.024%	…+	NNN+ [3+ bp INS]
13,435	1.9%	0.017%	T	A
13,215	1.9%	0.017%	A	T
11,925	1.7%	0.015%	T	G
11,617	1.6%	0.015%	A	C
9681	1.4%	0.013%	G	C
9524	1.3%	0.012%	C	A
9469	1.3%	0.012%	C	G
9442	1.3%	0.012%	G	T
4188	0.6%	0.005%	NN	.. [2 bp DEL]
3444	0.5%	0.004%	NNN+	…+ [3+ bp DEL]

Since in the current study, the Trinity [genome-guided]-based approach using RNA-seq data gave a marginally higher complement of USCO genes, we used this gene set for further analyses. In addition to an elevated proportion of fragmented USCOs (see below), we also noted a high proportion of duplicated USCOs. Inspection revealed that some of these were bona fide lineage-specific expansions. For example, the analysis uncovered three distinct loci encoding isoforms of fructose 1,6-bisphosphatase (PFAM: PF00316), as predicted also from the WTSI assembly. Pairs of USCOs were also found on homologous contigs. There were four such examples in the 12 heterozygous branch subgraphs alone; this presumably reflects haplotypic variants.

**Fig. 8:**
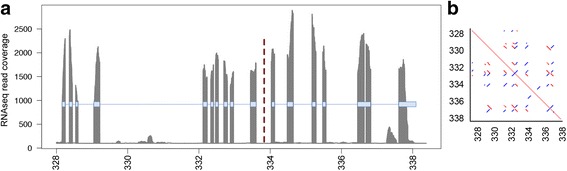
Repeat sequence confounds Trinity gene prediction. **a** CAZy analysis indicated that two adjacent Trinity-generated transcripts (c16_g1_i2 and c16_g1_i1, left and right, respectively; exons indicated by blue boxes) had been incorrectly predicted since they correspond to C- and N-terminal fragments of a single CAZyme gene (of the GT33 family). The read coverage of the exons for the two predicted transcripts was similar, supporting a single-gene model. BUSCO erroneously called c16_g1_i1 as complete (EOG091H03EM0). **b** A self-map dot plot of the genomic context in which these transcripts reside demonstrated a high degree of sequence similarity throughout the region, where the two predicted transcripts were split by a palindromic repeat motif that switched directions at the intersection of the two transcripts. The coordinates (in kbp) on the contig tig000206 are shown

While generating a complete high-quality annotation was beyond the scope of this study, we made use of expert knowledge regarding carbohydrate-active enzymes (CAZymes) to provide a complementary insight into the predicted genes compared to the WTSI set [29]. Of the 62 different domain architectures among the 158 well-predicted CAZyme proteins from our assembly, only 36 were represented among the set of 96 such proteins in the WTSI reference proteome. Our assembly included a further 16 domain architectures represented among proteins that were flagged as being incorrectly predicted (N- or C-terminal fragments; Additional file 5: Table S3). Manual inspection revealed that in most cases, this was a consequence of the presence of non-tandem repeats (of which predicted transposable elements were a subset), where the repetition created ambiguity for transcript assembly by Trinity (Fig. 8). These structures, which also contributed to the presence of the fragmented USCOs mentioned above, were even more of a problem for gene prediction in the WTSI assembly; they not only contributed to the fragmentation of USCOs and CAZymes (Additional file 5: Table S3), but also introduced contig breaks that could not be bridged in the absence of long reads.

As explained above, during the long-term culture of *N. brasiliensis*, attempts are made to maintain genotypic diversity in the population. The assembly that we produced reflects this. Surgical transplantation of single adult parasitic worms has very recently been shown to be feasible, allowing controlled matings and the establishment of inbred lines [30]. A less demanding alternative approach to describe the different genotypes within a population would be to assemble separate genome sequences directly from DNA extracted from individual nematodes. Current sequencing technologies are not sufficiently sensitive to allow this. Whole-genome amplification (WGA) was, therefore, used to generate sufficient DNA from a single adult worm. This was then sequenced with a single MinION run. Base-calling using Albacore produced 2,074,871 reads for 6.5 Gb of sequence (compared to 1,722,835 reads totaling 4.9 Gb of sequence using the older MinKNOW approach). Based on the initial FASTQ files, the 90th percentile of length for the WGA sequencing run was 6.4 kb, with more than 27,000 reads of at least 13 kb. The overall read length distribution for the WGA run was somewhat shifted to smaller sizes (Additional file 6: Figure S3), presumably a consequence of Phi29 processivity.

These reads were fed into Canu v1.5, which automatically adjusts assembly parameters when read coverage is low, giving an unpolished assembly of 3280 contigs and 119 Mb of assembled genomic sequence (Table 1). WGA methods can suffer from chimera formation and amplification bias. The proportion of WGA reads that mapped over 90% of their length to the WGA assembly was lower than that of WGA reads mapping to the final assembly (41.9% vs. 53.3%; Table 5), with a similar distribution of mapped read lengths, indicating that the WGA process did not produce significantly more chimeric reads than that for the unamplified DNA. Of note, any randomly combined chimeric sequences would be clipped by Canu’s assembly process due to the absence of support for the chimeric structure. On the other hand, we did observe a higher than expected proportion of reads that mapped along close to 50% of their length to the target genome. Some of these reads may be template/complement read pairs that have been inappropriately combined by the software due to fast pore loading of the DNA fragments. To investigate this effect in the future, reads that map partially to contigs wherein the contig ends before the read ends would need to be filtered out; we did not do this in the current study.

**Table 5:** Read mapping statistics

Read category	WGA		Unamplified	
Assembly	WGA	Final	WGA	Final
Read count	1,362,775	1,362,775	2,137,475	2,137,475
Mapped	1,106,671	1,233,514	1,525,846	1,922,031
Well mapped^1^	571,180	727,433	563,302	1,386,713
Uniquely mapped^2^	16,977	143,820	16,568	412,753
Median mapped read proportion^3^	91.1%	94.1%	65.6%	96.7%
Median mapped proportion excluding well-mapped reads	56.1%	65.9%	31.1%	67.2%

The number of reads that were generated from the WGA DNA and that mapped to either the assembly made from those reads (WGA) or from those generated from unamplified DNA (Final) are shown in columns 2 and 3. The number of reads that were generated from unamplified DNA (Unamplified) and that mapped to either the assembly made from the WGA reads (WGA), or from those generated from unamplified DNA (Final) are shown in columns 4 and 5. Since a similar fraction of WGA and unamplified reads do not map to the final assembly (approximately 10%), it appears that contaminating DNA was effectively removed (see ‘Methods’ for details). While the figure for the mapping of reads from the unamplified DNA to the WGA assembly is comparatively low, it is higher than the equivalent comparison of the completeness of genomes (100 × [completeness WGA/completeness final]), which is closer to 55% for Nanopolished assemblies (not shown), or 32.4% by stringent pairwise mapping. The difference is explained by redundant mapping of haplotype-specific and repeat-containing reads from the unamplified DNA

*WGA* whole-genome amplification

^1^Reads that map at least 90% of their length to the reference assembly

^2^Reads that map only to the target assembly, and not the other assembly

^3^Median proportion of individual reads that aligned to the reference assembly

Since the WGA reads that mapped only to the final assembly (in regions of low sequence coverage) represented 11% of the total number, sequencing depth was not a major limiting factor. As the stringent pairwise mapping also indicated that the WGA assembly captured a third (32.4%) of the predicted genome, it appears that genome amplification was indeed biased; future attempts should use alternate amplification (e.g. primer-free) approaches. The results do, however, illustrate that assembling a complete genome from a single nematode is within the bounds of current technology and this method could be used to survey genotypic diversity in populations.

## Conclusions

For researchers interested only in coding genes and their expression, standard short-read RNA-seq will continue to be an efficient and appropriate technology. As we showed here, however, Illumina-type DNA sequencing is particularly poor as a basis for assembling genomes rich in long repeats. On the other hand, our results illustrate that a de novo assembly of high quality can be obtained using only long reads, even of a complex genome from a heterogeneous population, using a very modest sequencing depth (24× after trimming and correction). At the local level, the incorporation of sequencing polishing, using only MinION reads, raises sequence quality close to that seen with Illumina-based approaches. The results revealed the need for further improvements in resolving ambiguous contig architectures and in transcript-directed gene structure prediction. Nevertheless, since haplotypic variation could be detected even without RNA-seq-directed sequence correction, they clearly show the potential for using this approach to profile parasite populations, opening the way for detailed molecular epidemiological studies.

## Methods

### Canu 1.5

Changes to Canu relative to v1.4 are documented in the v1.5 release notes (available at https://github.com/marbl/canu/releases/tag/v1.5). Briefly, one major change was a switch in the alignment algorithm used to generate consensus for the corrected reads and final contigs. This improved the corrected reads by slightly increasing their identity and splitting fewer reads. Canu also started using the raw read overlaps to estimate corrected read lengths and used that information to inform its selection of reads for correction. As with all such analysis packages, bugs continue to be identified and corrected. The first release of v1.5, for example, included a bug that erroneously removed heterozygous edges such that in the type of subgraphs shown in Fig. 4a, the results presented would potentially correspond to lower bounds of complexity. This and other issues have been corrected in recent development versions of Canu and the latest public release.

### Nematode cultures

The *N. brasiliensis* strain used in this study was originally sourced from Lindsey Dent (University of Adelaide) and maintained at the Malaghan Institute for 22 years by serial passage through female Lewis rats [31]. Ethics approval is overseen and approved by the Animal Ethics Committee of Victoria University of Wellington. The strain used for the WTSI reference genome is not fully documented. In principle, it derives from a line that had been maintained serially at the National Institute for Medical Research at Mill Hill by Bridget Ogilvie starting in the early 1970s and then by Rick Maizel, at Imperial College. Murray Selkirk continued to maintain the strain at Imperial after R. Maizel moved to the University of Edinburgh, where he established parallel lines. These Edinburgh lines were supplemented on a number of occasions with cultures from Imperial College. The standard cycle involved injection of 4000–6000 L3 larvae into Sprague–Dawley rats, but was otherwise similar to that used at the Malaghan (R. Maizel, personal communication).

### DNA preparation

Five samples of 25 mg each of frozen worms, cultured, harvested and purified as previously described [31], were sent to Simon Mayes (ONT) and subject to different methods of sample preparation (see Additional file 4: Table S2):

1. Sonication, DNeasy: Worms were disrupted by sonication and then processed using a Qiagen DNeasy kit (ATL/AL, 30 min at 56 °C). This yielded 900 ng of DNA and 1800 ng of RNA.
2. Sonication, G2, Tip20: Worms were disrupted by sonication, then lysed using Qiagen buffer G2 (30 min at 56 °C). The lysate was purified using a Qiatip 20 anion exchange column. This yielded 800 ng of DNA.
3. Direct lysis, DNeasy: Intact worms were added without prior disruption directly into Qiagen buffer G2 (30 mins at 56 °C). The lysate was processed using a Qiagen DNeasy kit (ATL/AL, 30 min at 56 °C). This yielded 700 ng of DNA and 17,700 ng of RNA.
4. Pipette squashed, G2, Tip20: Worms were mashed against the side of the sample tube using a pipette tip. The cells then underwent chemical lysis in the Qiagen buffer G2 (120 min at 56 °C) and lysate was purified using the Qiatip 20 anion exchange column. The DNA was fragmented using a Covaris G-tube prior to library preparation. This yielded 1100 ng of DNA.

All samples were subsequently processed using the ONT 1D Ligation Sequencing Kit (SQK-LSK108), ligating the ONT adapter mix onto end-prepped and dA-tailed DNA. Each prepared library was loaded onto a different MinION flow cell for sequencing.

### Genome assembly

Only single sequencing runs were performed for each sample. The MinION is a random-access sequencer. Our previous detailed research on the *N. brasiliensis* mitochondrial genome [31] and current research on mouse cDNA (DE, unpublished) has demonstrated that coverage is surprisingly uniform. If the same sample were sequenced multiple times, there would be differences in the precise reads obtained, but the accumulated experience of the many labs using the MinION shows that it will not change the genomic distribution of reads nor the systematic errors in base-calling and consensus assembly in any significant way. Raw reads and called FASTQ files were obtained from ONT (called by workflow “1D RNN Basecalling 450 bps FastQ v1.121”) following sequencing. Base-calling was performed remotely at the time of sequencing by Metrichor. The specific base-calling version ID was reported as:

- chimaera version = 1.23.4
- dragonet version = 1.23.0
- event_detection = Analyses/EventDetection_000
- name = ONT Sequencing Workflow
- segmentation = Analyses/Segment_Linear_000
- time_stamp = 2017-Jan-21 07:55:13

The raw reads were recalled using Albacore 1.1.0, which implements a time-domain correction (transducer) for homopolymeric regions in the called sequence. This is also available in the open-source Scrappie (https://github.com/nanoporetech/scrappie) base caller:

for x in $(ls -d [CFED]_??????); do echo ${x}; read_fast5_basecaller.py -t 6 -i ${x} -s called_${x} -o fastq -c r94_450bps_linear.cfg; done

Illumina reads for the WTSI genome assembly were retrieved from the Sequence Read Archive (accession ERR063640). These reads were processed into k-mer counts using Jellyfish v2.2.6 [32], and the resulting histogram used in GenomeScope (see below):

jellyfish count -C --bf-size 20G -t 6 -s 2G -m 21 <(zcat ERR063640_1P.fastq.gz) <(zcat ERR063640_2P.fastq.gz) -o ERR063640_mer_counts.jf

jellyfish histo ERR063640_mer_counts.jf > ERR063640_mer_counts.histo

Using the FASTQ files, rather than just reads from the called pass bin, allowed a maximum amount of sequence information to be recovered. Reads were trimmed by 65 bp at each end to exclude adapters (see below), then Canu v1.5 was run with default parameters. The estimated genome size was 205 Mb as determined using GenomeScope (qb.cshl.edu/genomescope/) and the WTSI Illumina reads. Long-read sequencing of a heterogeneous population would be expected to generate a longer genome size due to the separation of haplotypes. We, therefore, made a conservative estimate of 300 Mb. The assumed genome size alters how many reads are selected for the final Canu assembly. If the coverage of corrected reads is greater than 40×, then only the longest reads are used (up to 40× coverage). In our case, the coverage was below 40× regardless of what parameters were used, so it would not be expected to alter the outcome. Within Canu, assembly parameters are adjusted when read counts are below 20× (e.g. corMinCoverage = 0) as previously described [33]:

## trim reads

pv called_[CFED]_*_albacore_1.1.0.fq.gz | zcat | ~/scripts/fastx-fetch.pl -t 65 | gzip > called_CFED_65bptrim_albacore_1.1.0.fq.gz

## run Canu

~/install/canu/canu-1.5/Linux-amd64/bin/canu -nanopore-raw called_CFED_65bptrim_albacore_1.1.0.fq.gz -p Nb_ONTCFED_65bpTrim_t1 -d Nb_ONTCFED_65bpTrim_t1 genomeSize=300 M

Bowtie2 was used in local mode to map RNA-seq reads to the assembled genome contigs:

bowtie2 -p 10 --local -x Nb_ONTCFED_65bpTrim_t1.contigs.fasta -1 ../1563-all_R1_trimmed.fastq.gz -2 ../1563-all_R2_trimmed.fastq.gz | samtools sort > 1563_vs_uncorrected_NOCFED.bam

Pilon was used to correct based on the RNA-seq mapping to the genome, with structural reassembly disabled (in case it collapsed introns):

java -Xmx40G -jar ~/install/pilon/pilon-1.22.jar --genome Nb_ONTCFED_65bpTrim_t1.contigs.fasta --frags 1563_vs_uncorrected_NOCFED.bam --fix snps,indels --output BT2Pilon_NOCFED --gapmargin 1 --mingap 10000000 --threads 10 --changes 2>BT2Pilon_NOCFED.stderr.txt 1>BT2Pilon_NOCFED.stdout.txt

Contigs that were entirely composed of homopolymer sequences were identified using grep and removed from the assembly:

## identify homopolymer (and binary division-rich) regions

pv BT2Pilon_NOCFED.fasta | ~/scripts/fastx-hplength.pl > hplength_BT2Pilon_NOCFED.txt

pv BT2Pilon_NOCFED.fasta | ~/scripts/fastx-hplength.pl -mode YR > hplength_YR_BT2Pilon_NOCFED.txt

pv BT2Pilon_NOCFED.fasta | ~/scripts/fastx-hplength.pl -mode SW > hplength_SW_BT2Pilon_NOCFED.txt

pv BT2Pilon_NOCFED.fasta | ~/scripts/fastx-hplength.pl -mode MK > hplength_MK_BT2Pilon_NOCFED.txt

## example grep hunt for repeated sequence

cat BT2Pilon_NOCFED.fasta | grep -e '^[AT]\{80\}' -e '^>' | grep --no-group-separator -B 1 '^[AT]\{80\}' | ~/scripts/fastx-length.pl

## exclude contigs and sort by length

~/scripts/fastx-fetch.pl -v tig00010453 tig00024413 tig00024414 tig00023947 | ~/scripts/fastx-sort.pl -l > Nb_ONTCFED_65bpTrim_t1.contigs.hpcleaned.fasta

This produced the final assembly described in the paper. At this stage, we had an assembled genome, but validation of the genome was difficult. We decided to carry out a draft genome-guided transcriptome assembly with Trinity. To evaluate the completeness of the genome, we focused on expressed genes and used a set of Illumina RNA-seq reads to perform a genome-guided transcriptome assembly using Trinity.

The RNA-seq reads were remapped to the corrected assembly for genome-guided Trinity:

bowtie2 -p 10 -t --local --score-min G,20,8 -p 10 -x BT2Pilon_NOCFED_hpcleaned.fasta --rf -X 15000 -1

../1563-all_R1_trimmed.fastq.gz -2 <(pv../1563-all_R2_trimmed.fastq.gz | zcat) 2>bowtie2_1563_vs_BNOCFED_hp.summary.txt | samtools sort > bowtie2_1563_vs_BNOCFED_hp.bam

## Trinity assembly; assume introns can be up to 15 kb in length

~/install/trinity/trinityrnaseq-Trinity-v2.4.0/Trinity --CPU 10 --genome_guided_bam bowtie2_1563_vs_BNOCFED_hp.bam --genome_guided_max_intron 15000 --max_memory 40G --SS_lib_type RF --output trinity_BNOCFED

The assembly that Trinity generated had similar completeness (as measured by BUSCO) to a de novo assembly generated using the same RNA-seq reads (see Table 3). The assembly was, however, very large, with over 350 k contigs (see Table 1), most likely due to isoform fragments being included in the transcriptome. We carried out additional filtering steps, reducing the number of assembled contigs while maintaining similar BUSCO completeness scores.

### Transcript filtering

Our RNA-seq data is publicly available from the European Nucleotide Archive of the European Bioinformatics Institute (https://www.ebi.ac.uk/ena/data/view/ERS1809079), where details of the samples can be found.

The estimated numbers of mapped RNA-seq reads (as predicted by Salmon [34]) were used to filter transcripts, because the genome-guided assembly was based on RNA-seq expression. Salmon uses a pseudo-alignment approach and was chosen to correct for reads mapping to the same sequence, but on different isoforms and/or genes rather than alternative alignment-based approaches that are designed around the expectation of a relatively complete and non-repetitive genome. Since this analysis was to estimate the expression distribution, however, the specific mapping strategy is not expected to influence greatly the outcome. RNA-seq reads were mapped to the Bowtie2/Pilon genome-guided assembly, and a threshold of 50 reads for true expression of complete BUSCO genes was chosen for filtering transcripts from the genome-guided Trinity transcriptome. The longest open reading frame from each transcript was extracted to generate a protein sequence for further collapsing using cdhit to remove protein sequences that had at least 98% identity to a longer protein, leaving 56,980 transcripts. Each of these steps are outlined below.

The RNA-seq reads were mapped to the Trinity-generated transcripts using Salmon:

## create Salmon index

~/install/salmon/Salmon-0.8.2_linux_x86_64/bin/salmon index -t Trinity-BNOCFED.fasta -i Trinity-BNOCFED.fasta.sai

## quantify transcript coverage with Salmon

~/install/salmon/Salmon-0.8.2_linux_x86_64/bin/salmon quant -i Trinity-BNOCFED.fasta.sai -1 ../../1563-all_R1_trimmed.fastq.gz -2 ../../1563-all_R2_trimmed.fastq.gz -p 10 -o quant/1563-all_quant -l A

The expression of BUSCO genes was used to set a credible signal cutoff (Additional file 7: Figure S4). A threshold of 50 mapped reads was chosen, as 98% of complete BUSCO sequences had more than 50 mapped reads. The transcripts were subsetted based on their expression scores:

## Subset transcripts based on a threshold of 50 counts

pv Trinity-GG.fasta | ~/scripts/fastx-fetch.pl -i HighCount_Transcripts_Num50.txt > HighCount50_TBNOCFED.fasta

The longest Met to Stop open reading frame was identified for each transcript for protein-based clustering with cdhit:

pv HighCount50_TBNOCFED.fasta | getorf -find 1 -noreverse -sequence/dev/stdin -outseq/dev/stdout | ~/scripts/fastx-isofilter.pl -o > longest_MetStopORF_HC50_TBNOCFED.fasta

## run cdhit

cdhit -T 10 -c 0.98 -i longest_MetStopORF_HC50_TBNOCFED.fasta -o cdhit_0.98_LMOHC50_TBNOCFED.prot.fasta

## identify names of longest representative proteins for each cluster

grep '^>' cdhit_0.98_LMOHC50_TBNOCFED.prot.fasta | perl -pe 's/^>//' > cdhit_0.98_LMOHC50_TBNOCFED.prot.names.txt

## fetch transcripts associated with the representative proteins

pv HighCount50_TBNOCFED.fasta | ~/scripts/fastx-fetch.pl -i cdhit_0.98_LMOHC50_TBNOCFED.prot.names.txt > cdhit_0.98_LMOHC50_TBNOCFED.tran.fasta

The longest isoform for each gene was identified, producing an isoform-collapsed transcriptome subset, which was clustered at the protein level by cdhit at 90% identity:

## extract longest protein for each transcript

cat cdhit_0.98_LMOHC50_TBNOCFED.prot.fasta | ~/scripts/fastx-isofilter.pl > LI_CD98LMOHC50_TBNOCFED.prot.fasta

## cluster at 90% via CDHIT

cdhit -i LI_CD98LMOHC50_TBNOCFED.prot.fasta -o CDLI_CD98LMOHC50_TBNOCFED.prot.fasta

## find associated transcripts

grep '^>' CDLI_CD98LMOHC50_TBNOCFED.prot.fasta | perl -pe 's/^>//' > CDLI_CD98LMOHC50_TBNOCFED.names.txt

cat cdhit_0.98_LMOHC50_TBNOCFED.tran.fasta | ~/scripts/fastx-fetch.pl -i CDLI_CD98LMOHC50_TBNOCFED.names.txt > CDLI_CD98LMOHC50_TBNOCFED.tran.fasta

BUSCO was run on the collapsed transcripts to provide one measure of genome completeness:

## run BUSCO on isoform-collapsed transcripts in long genome mode

python ~/install/busco/BUSCO.py -i../CDLI_CD98LMOHC50_TBNOCFED.tran.fasta -o BUSCO_longgeno_CDLI_CD98LMOHC50_TBNOCFED_nematodes -l \

~/install/busco/nematoda_odb9 -m geno -c 10 --long

## run BUSCO on isoform-collapsed transcripts in transcript mode

python ~/install/busco/BUSCO.py -i../CDLI_CD98LMOHC50_TBNOCFED.tran.fasta -o BUSCO_tran_CDLI_CD98LMOHC50_TBNOCFED_nematodes -l \

~/install/busco/nematoda_odb9 -m tran -c 10

This filtered set had very similar BUSCO scores to the original genome-guided Trinity assembly, despite the number of transcripts being reduced down to 1/6 of their original count, and the length of the transcriptome being reduced down to less than 1/3 of its original size. The number of duplicated BUSCO genes in the filtered set suggests that this set could probably be made smaller with a looser cd-hit-est clustering, although there is a chance that such a reduction may cause gene copies to be clustered together.

The missing USCOs were remapped to our assembled genome to determine whether any of the corresponding genes were actually present in the genome:

blastx -num_threads 10 -query <(pv/mnt/gg_nanopore/gringer/ONT_Jan17/Nb_ONTCFED_65bpTrim_t1/Nb_ONTCFED_65bpTrim_t1.contigs.fasta)

-db missing_busco_list_intersectionBUSCO_nematodes.fasta -outfmt 6 -evalue 1e-3 > BLAST_NOCFED_vs_BUSCO_missing_intersection.tsv

Hits for the same contig/USCO combination were merged using a custom script, and sequence extracted from the assembly to cover the entire matched region:

(~/scripts/blastx_boundaries.pl BLAST_NOCFED_vs_BUSCO_missing_intersection.tsv | while read a b; do samtools faidx/mnt/gg_nanopore/gringer/ONT_Jan17/Nb_ONTCFED_65bpTrim_t1/Nb_ONTCFED_65bpTrim_t1.contigs.fasta ${b} | perl -pe "s/^>/>${a}-/"; done) > BLAST_NOCFED_vs_BUSCO_missing_intersection.fasta

### Whole-genome amplification and assembly

DNA extracted from a single adult worm was amplified using a Qiagen Midi RepliG kit. Raw reads and called FASTQ files were obtained from ONT (called by workflow “1D RNN Basecalling 450 bps FastQ v1.121”) following sequencing. Reads were filtered to exclude those from contaminating DNA using OneCodex (http://onecodex.com). Reads that mapped to any DNA in the OneCodex database were excluded from the read set:

(zcat OneCodex_RefSeq_132394.fastq.gz.results.tsv.gz | awk '{if($3 == 0){print $1}}'; zcat OneCodex_OCD_132394.fastq.gz.results.tsv.gz | awk '{if($3 == 0){print $1}}') | sort | uniq -d | gzip > OCunmapped_names_132394.txt.gz

pv 132394.fastq.gz | zcat | ~/scripts/fastx-fetch.pl -i OCunmapped_names_132394.txt.gz | ~/scripts/fastx-fetch.pl -v -i ONTmapped_names_132394.txt.gz | gzip > OCunmapped_ONTunmapped_132394.fastq.gz

Filtered reads from the WGA sample were called using Albacore 1.1.0:

read_fast5_basecaller.py -o fastq -i A_132394 -t 10 -s called_A_132394 -c r94_450bps_linear.cfg

Reads with a length of greater than 10 k were extracted for subsequent analysis:

pv called_A_132394_albacore_1.1.0.fq.gz | zcat | ~/scripts/fastx-fetch.pl --min 10000 | gzip > 10k_called_A_132394_albacore_1.1.0.fq.gz

To define the region of raw nanopore sequences for adapter exclusion, the >10 k reads were mapped to 50 M reads that had been generated by WTSI and that had been used for the existing WTSI assembly:

bowtie2 -p 10 --no-unal --no-mixed --local -x 10k_called_A_132394_albacore_1.1.0.fa -1 <(pv ~/bioinf/MIMR-2017-Jan-01-GBIS/GLG/ONT/aws/Sampled_50M_ERR063640.R1.fq.gz | zcat) -2 ~/bioinf/MIMR-2017-Jan-01-GBIS/GLG/ONT/aws/Sampled_50M_ERR063640.R2.fq.gz | samtools sort > WTSI_Sampled_50M_vs_10k_called_A_132394.bam

## find position of first mapped Illumina read for each nanopore read

pv WTSI_Sampled_50M_vs_10k_called_A_132394.bam | samtools view - | awk '{print $3,$4}' | sort -k 1,1 -k 2,2n | sort -u -k 1,1 | gzip > firstHit_WTSI_Sampled_50M_vs_10k_called_A_132394.txt.gz

## determine position of last mapped Illumina read

pv WTSI_Sampled_50M_vs_10k_called_A_132394.bam | samtools view - | awk '{print $3,$4}' | sort -k 1,1 -k 2,2rn | sort -u -k 1,1 | gzip > lastHit_WTSI_Sampled_50M_vs_10k_called_A_132394.txt.gz

## count positions of first reads

zcat firstHit_WTSI_Sampled_50M_vs_10k_called_A_132394.txt.gz | awk '{print $2}' | sort -n | uniq -c | gzip > firstBase_counts_WTSI_Sampled_50M_vs_10k_called_A_132394.txt.gz

When we mapped the 5' ends of Illumina reads to the start of nanopore reads with Bowtie2, there was a common register shift of 28–32 bases, corresponding to the presence of adapter sequences in the nanopore reads. Reads were conservatively trimmed by 65 bases at each end to exclude adapters:

pv called_A_132394_albacore_1.1.0.fq.gz | zcat | ~/scripts/fastx-fetch.pl --min 1130 --max 1000000 | \

~/scripts/fastx-fetch.pl -t 65 | gzip > 65bpTrim_called_A_132394_albacore_1.1.0.fq.gz

Canu v1.5 was used to assemble the trimmed reads. The assembly was done in stages (with an assembly at each stage) to determine whether or not particular stages were redundant for the assembly:

## attempt assembly-only with Canu v1.5

~/install/canu/canu-1.5/Linux-amd64/bin/canu -assemble -nanopore-raw 65bpTrim_called_A_132394_albacore_1.1.0.fq.gz -p Nb_ONTA_65bpTrim_t1 -d

Nb_ONTA_65bpTrim_t1 genomeSize=300 M

## attempt assembly + correction

~/install/canu/canu-1.5/Linux-amd64/bin/canu -assemble -nanopore-corrected 65bpTrim_called_A_132394_albacore_1.1.0.fq.gz -p Nb_ONTA_65bpTrim_t2 -d Nb_ONTA_65bpTrim_t2 -correct genomeSize=300 M

## attempt stringent trim with corrected reads

~/install/canu/canu-1.5/Linux-amd64/bin/canu -trim-assemble -p Nb_ONTA_65bpTrim_t3 -d Nb_ONTA_65bpTrim_t3 genomeSize=300 M -nanopore-corrected Nb_ONTA_65bpTrim_t2/Nb_ONTA_65bpTrim_t2.correctedReads.fasta.gz -trim-assemble trimReadsOverlap=500 trimReadsCoverage=5 obtErrorRate=0.25

An alternative, less-stringent overlap was also attempted (with trimReadsCoverage = 2), but resulted in a less complete assembly. The results of an analysis surrounding the different assemblies suggested that the default Canu assembly process of correction, trimming, then assembly produced the best outcome, namely the WGA assembly presented in Table 1.

Canu includes a step of normalization, reducing the shortest reads, if coverage is over a predefined threshold, but that normalization threshold was not triggered for our assembly. We did not attempt a k-mer-based normalization, since this would not resolve the incomplete genome sequence coverage. Selective amplification combined with random sampling by the sequencer means that poorly amplified regions were unlikely to be present in the sequenced reads. We did notice that an assembly combining both WGA and unamplified DNA samples produced a more fragmented genome, which is why a combined assembly is not presented here. It is possible that k-mer normalization of the WGA reads might improve such a hybrid assembly.

## Additional files

Additional file 1: Table S1. Detailed comparative sequence analysis and genome assembly statistics. (DOCX 12 kb)

Additional file 2: Figure S1. Dot plots of all-against all sequence comparisons between the five most compressible VeCTR regions, based on minimum-distance alignments using LAST-align, created using LAST-dotplot. The names of the corresponding contigs and the coordinates of the plotted regions are indicated for each VeCTR and their unique flanking sequences. The longest of these five VeCTRs corresponds to 147 repeats of tRNA-Trp followed by 114 bp of non-conserved sequence, while the shortest contains 90 copies of tRNA-Ser with 80 bp of non-conserved sequence. (PDF 69 kb)

Additional file 3: Figure S2. Potential orthologs of missing USCOs. **a** Venn diagram showing the overlap for USCOs flagged as missing in the analysis of three genome assemblies (Uncorrected (Canu only), Nanopolish and Trinity [genome-guided]; see Table 3). Among the 34 common missing USCOs listed here, several have credible orthologs in the assembled genomes. (B-E) Examples of two putative orthologs for “missing” USCOs, showing the dot matrix view of an NCBI tblastn-2sequences search between the indicated USCO and region of a contig from the Trinity [genome-guided] assembly (**b**,**d**) and the sequence alignment, generated using Muscle, with the predicted *N. brasiliensis* protein sequence extracted from the tblastn alignment (**c**,**e**). For both *N. brasiliensis* sequences, note the presence of stop codons (*) in the sequence; these will confound BUSCO analysis. (PDF 398 kb)

Additional file 4: Table S2. BLAST hits for missing USCOs (see Excel file). (XLSX 78 kb)

Additional file 5: Table S3. CAZy analysis (see Excel file). (XLSX 265 kb)

Additional file 6: Figure S3. Distribution of read lengths for whole-genome amplified (WGA) DNA. Comparison of the distribution of read lengths for amplified DNA (WGA) and unamplified DNA extracted by method 1 (see Fig. 2). (PDF 131 kb)

Additional file 7: Figure S4. The distribution of the number of mapped reads for BUSCO sequences. The area in gray on the left-hand graph is shown enlarged on the right. (PDF 147 kb)
