# Supplementary material for: De novo assembly of the complex genome of Nippostrongylus brasiliensis using MinION long reads
Source: BMC Biol. 2018 Jan 11;16:6. doi: 10.1186/s12915-017-0473-4 (PMC5765664; doi:10.1186/s12915-017-0473-4)
Supplement: Supplementary file 1 — Detailed comparative sequence analysis and genome assembly statistics. (DOCX 12 kb) [file 12915_2017_473_MOESM1_ESM.docx]

| Sample/Method^#^ | Number reads* | Total (Mb) | Long-est (kb)** | Mean (kb) | Median (kb) | N10 (kb) [N10] | N50 (kb) [N50] | N90 (kb) [N90] |
| --- | --- | --- | --- | --- | --- | --- | --- | --- |
| 1/M | 930281 | 2375 | 178.7 | 2.6 | 1.1 | 19.0 [9419] | 5.6 [109080] | 1.0 [518972] |
| 1/A | 922376 | 2597 | 201.0 | 2.8 | 1.2 | 21.8 [8418] | 6.3 [105646] | 1.0 [508748] |
| 2/M | 1571846 | 2290 | 164.2 | 1.5 | 0.7 | 40.6 [4260] | 2.3 [143757] | 0.6 [1039484] |
| 2/A | 1808087 | 3002 | 258.4 | 1.7 | 0.7 | 59.4 [3182] | 3.3 [117160] | 0.6 [1138477] |
| 3/M | 451065 | 1723 | 178.7 | 3.8 | 1.9 | 22.6 [5707] | 7.8 [62159] | 1.7 [242122] |
| 3/A | 445963 | 2028 | 233.9 | 4.5 | 2.1 | 40.6 [2412] | 9.5 [53732] | 1.9 [232972] |
| 4/M | 1958001 | 3834 | 86.6 | 2.0 | 0.7 | 11.8 [26826] | 5.7 [214829] | 0.7 [1075168] |
| 4/A | 2296453 | 4678 | 236.4 | 2.0 | 0.7 | 12.8 [27176 ] | 6.0 [243496] | 0.7 [1224833] |

**Additional file 1: Table S1. Detailed comparative sequence analysis and genome assembly statistics.**

^#^ The results are shown for MinKNOW/Canu v1.4 (M) or Albacore 1.1.0/Canu v1.5 (A) analysis of DNA reads for samples prepared by one of 4 methods (1-4; see **Table 2**).

* After quality control.

**A handful of unusally long sequences (up to several Mb) were filtered out, to retain only sequences of 265 kb or less. In a typical MinION run, there are a few of these erroneous reads that are caused by incorrect raw signal boundaries being set by the base calling software.
