# Supplementary figures and images for: De novo assembly of the complex genome of Nippostrongylus brasiliensis using MinION long reads
Source: BMC Biol. 2018 Jan 11;16:6. doi: 10.1186/s12915-017-0473-4 (PMC5765664; doi:10.1186/s12915-017-0473-4)

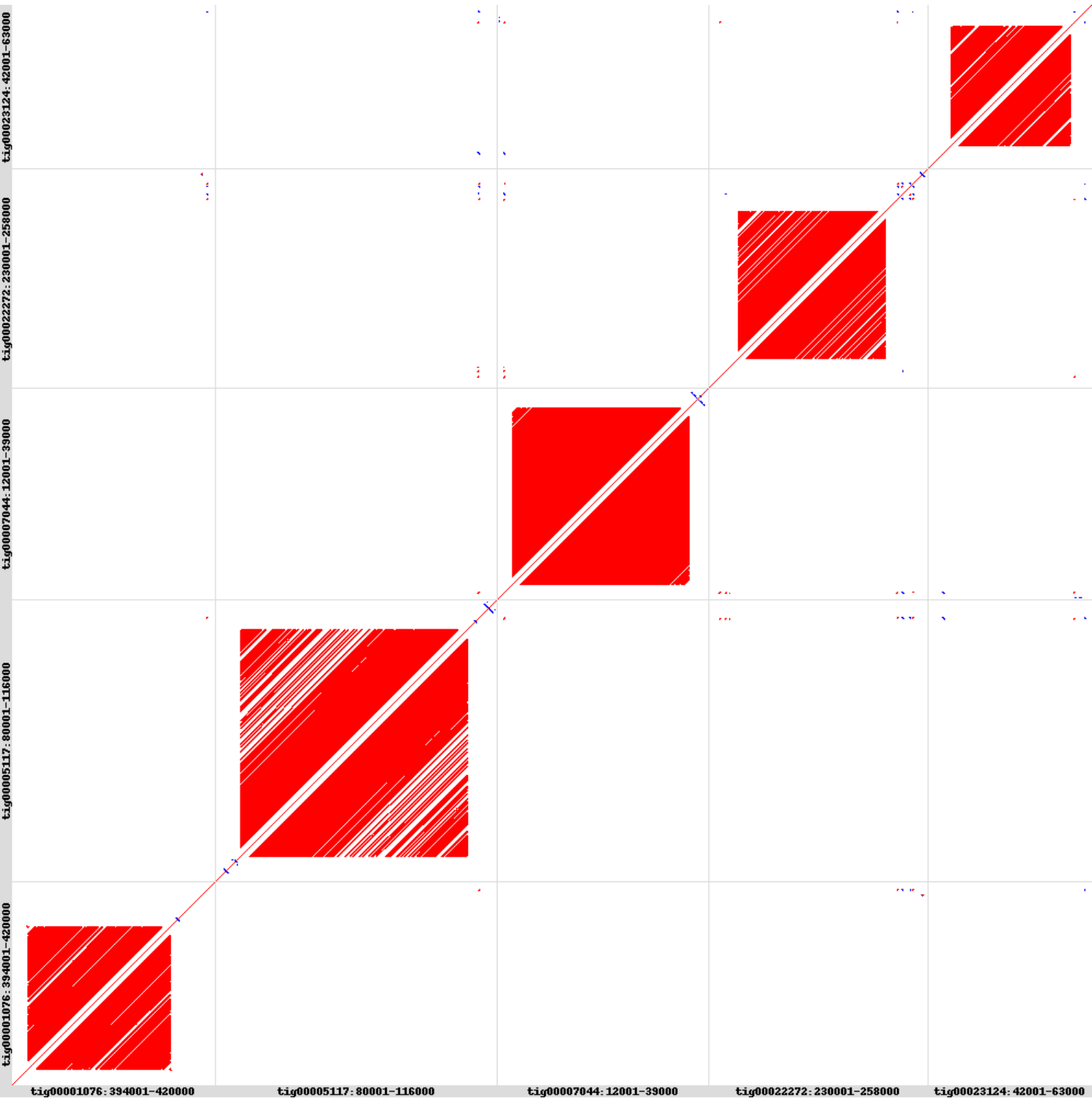

Supplement: Supplementary file 2 — Dot plots of all-against all sequence comparisons between the five most compressible VeCTR regions, based on minimum-distance alignments using LAST-align, created using LAST-dotplot. The names of the corresponding contigs and the coordinates of the plotted regions are indicated for each VeCTR and their unique flanking sequences. The longest of these five VeCTRs corresponds to 147 repeats of tRNA-Trp followed by 114 bp of non-conserved sequence, while the shortest contains 90 copies of tRNA-Ser with 80 bp of non-conserved sequence. (PDF 69 kb) [file 12915_2017_473_MOESM2_ESM.pdf]

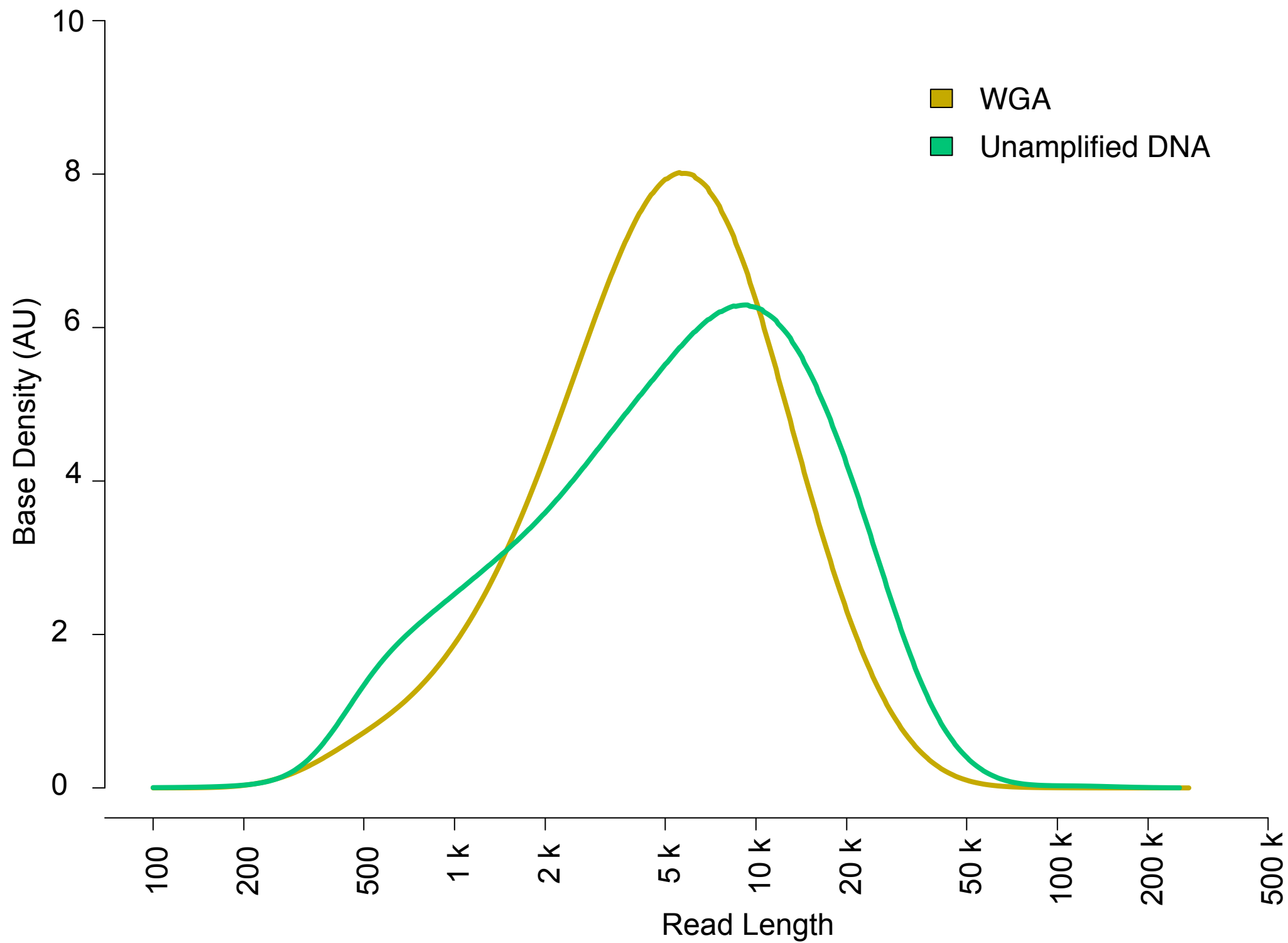

Supplement: Supplementary file 6 — Distribution of read lengths for whole-genome amplified (WGA) DNA. Comparison of the distribution of read lengths for amplified DNA (WGA) and unamplified DNA extracted by method 1 (see Fig. 2). (PDF 131 kb) [file 12915_2017_473_MOESM6_ESM.pdf]

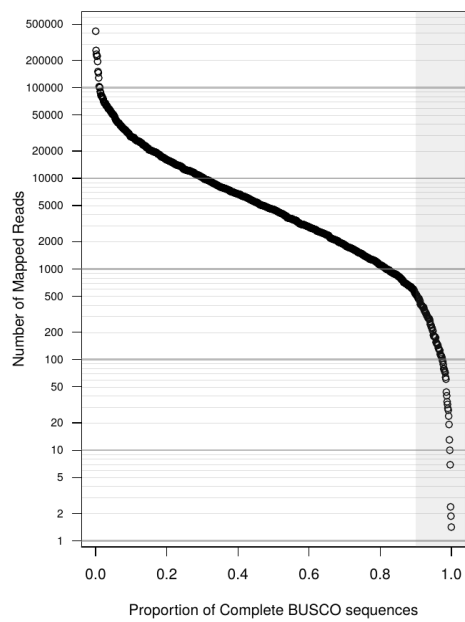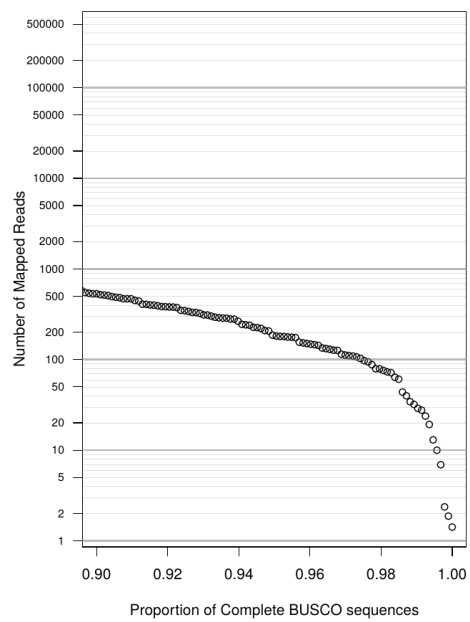

Supplement: Supplementary file 7 — The distribution of the number of mapped reads for BUSCO sequences. The area in gray on the left-hand graph is shown enlarged on the right. (PDF 147 kb) [file 12915_2017_473_MOESM7_ESM.pdf]
