# Supplementary material for: De novo assembly of the complex genome of Nippostrongylus brasiliensis using MinION long reads
Source: BMC Biol. 2018 Jan 11;16:6. doi: 10.1186/s12915-017-0473-4 (PMC5765664; doi:10.1186/s12915-017-0473-4)
Supplement: Supplementary file 3 — Potential orthologs of missing USCOs. a Venn diagram showing the overlap for USCOs flagged as missing in the analysis of three genome assemblies (Uncorrected (Canu only), Nanopolish and Trinity [genome-guided]; see Table 3). Among the 34 common missing USCOs listed here, several have credible orthologs in the assembled genomes. (B-E) Examples of two putative orthologs for “missing” USCOs, showing the dot matrix view of an NCBI tblastn-2sequences search between the indicated USCO and region of a contig from the Trinity [genome-guided] assembly (b,d) and the sequence alignment, generated using Muscle, with the predicted N. brasiliensis protein sequence extracted from the tblastn alignment (c,e). For both N. brasiliensis sequences, note the presence of stop codons (*) in the sequence; these will confound BUSCO analysis. (PDF 398 kb) [file 12915_2017_473_MOESM3_ESM.pdf]

A

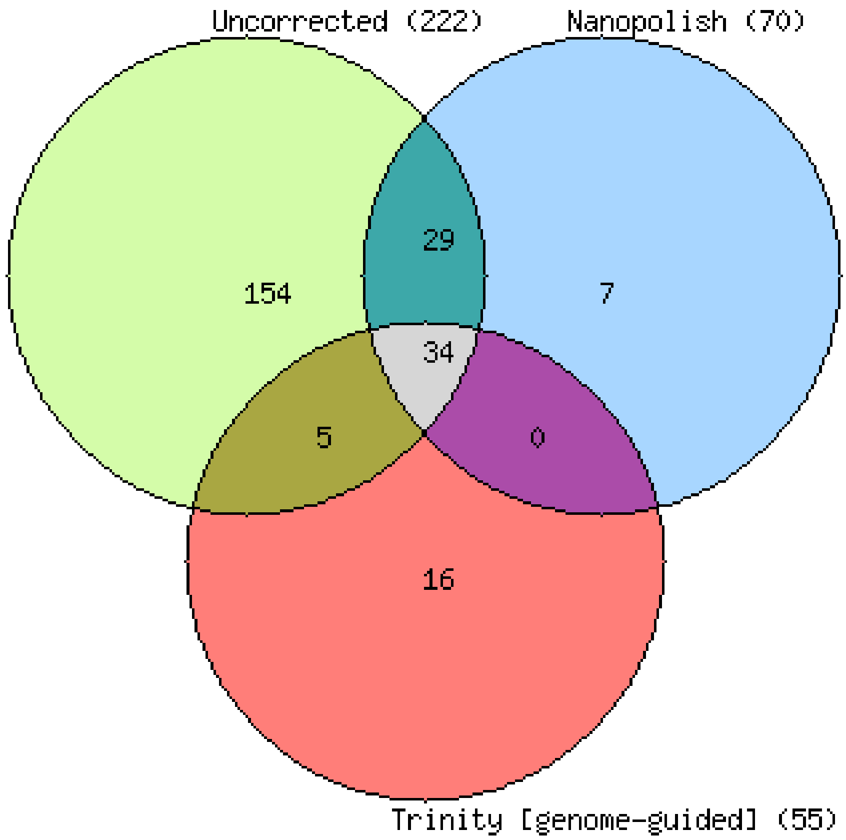

EOG091H004U	EOG091H01GP	EOG091H02G0	EOG091H03WO	EOG091H09UD
EOG091H00FN	EOG091H01MG	EOG091H02GU	EOG091H04PD	EOG091H0ARC
EOG091H00OW	EOG091H01MY	EOG091H02OK	EOG091H057W	
EOG091H00T3	EOG091H01R8	EOG091H02S6	EOG091H05HW	
EOG091H013W	EOG091H01SM	EOG091H037A	EOG091H05PM	
EOG091H017K	EOG091H01ZL	EOG091H039O	EOG091H07FE	
EOG091H01CQ	EOG091H028T	EOG091H03IC	EOG091H07LN	
EOG091H01DL	EOG091H02DM	EOG091H03U2	EOG091H08DZ	

B

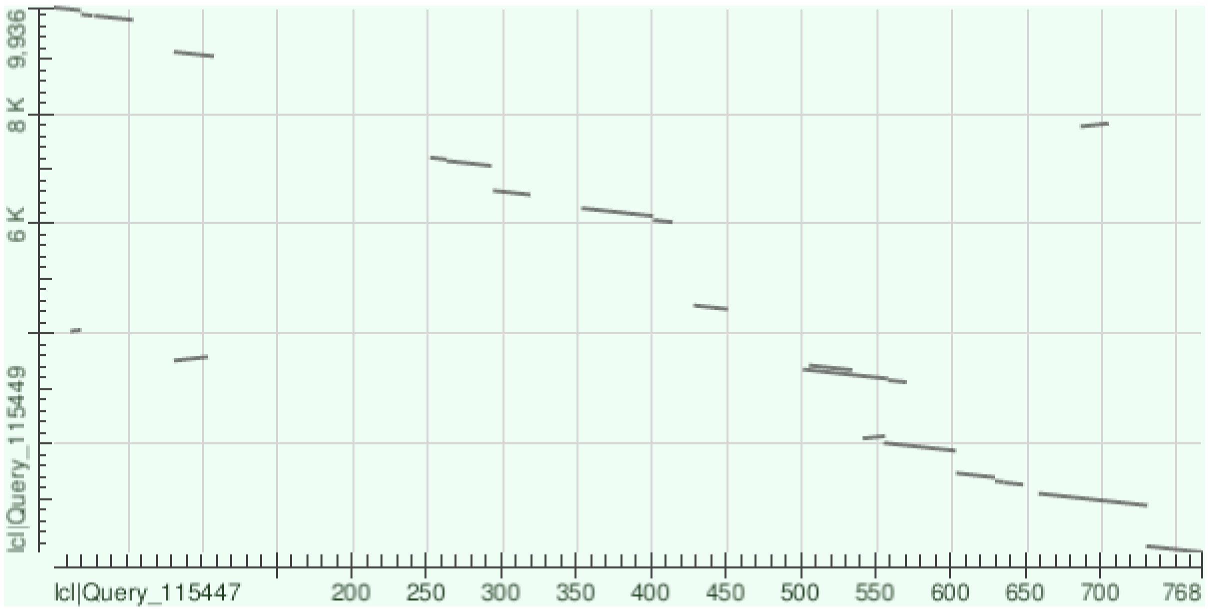

C

```
               1
tig00000853    -LVAVTNALFEDQVGKFDWRLQYVGCPNEVHFDTSG-KEDRLLVSTRENVFASL------------------------
EOG091H00T3    ALLASVSAIYEDQIGKFDWRKELVGCPKELKLEKKGSKSDRILVSTEESILASLKLNTGKIAWRKILESSSTSFSSDE

              79
tig00000853    ---FTVSDSGRVVRAWNKRNGALTWQWSIA---------------------------------------RLEFAIVDI
EOG091H00T3    ELLITVSKDGRVVRAWEKDNGVLVREVEISEEILVSDDKVFILEGAKLTAFSAIGSSEELIVVSGVEGKKLELIKIDA

             157
tig00000853    --------SKQLIVSEWV------------------------------------------------------------
EOG091H00T3    SLSVESFESKRCVVSEALLSCLSNKGLLVVKVELKSEIGEVIVVRTVSELEVYEITLSEAENEESLLVVASSNVVVVD

             235
tig00000853    ----------------------------REEALARISSVEMVDLPLSELQQMIEDEFE-ENIMSSFIRRLISQISQIH
EOG091H00T3    QAEKVSKEFEIVVVLEDCRLELLTVDESSEEALAKIASVELVDLPLSEAQASIESEFEDEDILEAFIRRLRSQIEQLK

             313
tig00000853    KWLLNTI----------------------------------DFFNLRKIIVATSLDGVVYGLDSSDGTIVWRLWLGSN
EOG091H00T3    RAIIEQILFVSSSLSFESKGLADFIASLRSSSSKKSEPLERDYFNLRKVIVVSTLKGVVFGIDSSDGSVLWKLYLGEN

             391
tig00000853    FSPLISSIGYQEQVPLFVQRTTAHY--------------LIFFNPITGKIVEQTQLPYPISR----------------
EOG091H00T3    FKPLESSLE-KEKVPLFIQRTTAHYATVVASDKKSEEAVLVSFNPITGKVVERKELATSVKRVELLPQKHVYPLLLVD

             469
tig00000853    ----------------------------------DIVSYQFSLKSRFVLFIAGNSAAHQ*KFCLEKVHSQGRVLIDRN
EOG091H00T3    KENKVKLYPELEATKPLSLLKLDRKSGALEGLELDVVKLSKTWKGNLSLESDEEIVAVKGKSLEQKVHSQGRVLVNRS

             547
tig00000853    VQYKYINPNLVAMATLDSEHQYLSLFLIDVVSGQMIHSARLAKATAPVHLVHCEHWIA-SYWSEKGRRTEIGVLELYE
EOG091H00T3    VLYKYVNPNLVAIAVLDKVEQELTITLVDAVSGQIVYSAKIKKIAGPVHLVHCENWIAYSYWSEKGRRVELGVVELYE

             625
tig00000853    GSEQTNVFDSLALLGVPEILTQSYIYAQ----------EKGLTTRSLLIALPLGGIHEVTRKLLDATRPLELTQEMRE
EOG091H00T3    GEEETDAFDSKL---KPVVLSQSYIFAQGVEALGVSETEKGLTTRSLLLALPFGGIYEVSKRLLDARRPLELTQELRE

             703
tig00000853    EMMIPYIPEIPIATEDMVNYNQTVHGVRGIKTA---------------------------------------------
EOG091H00T3    EMLIPYVPEIPIATEDLINYNQTVHSVRGIKTSPSGLESTSLVLAYGTDLFFTRLTPSGTFDILKDDFDHLLISLVLV

             781
tig00000853    ---------------------
EOG091H00T3    GLVVGSLVSKRLAKNNALKQA
```

D

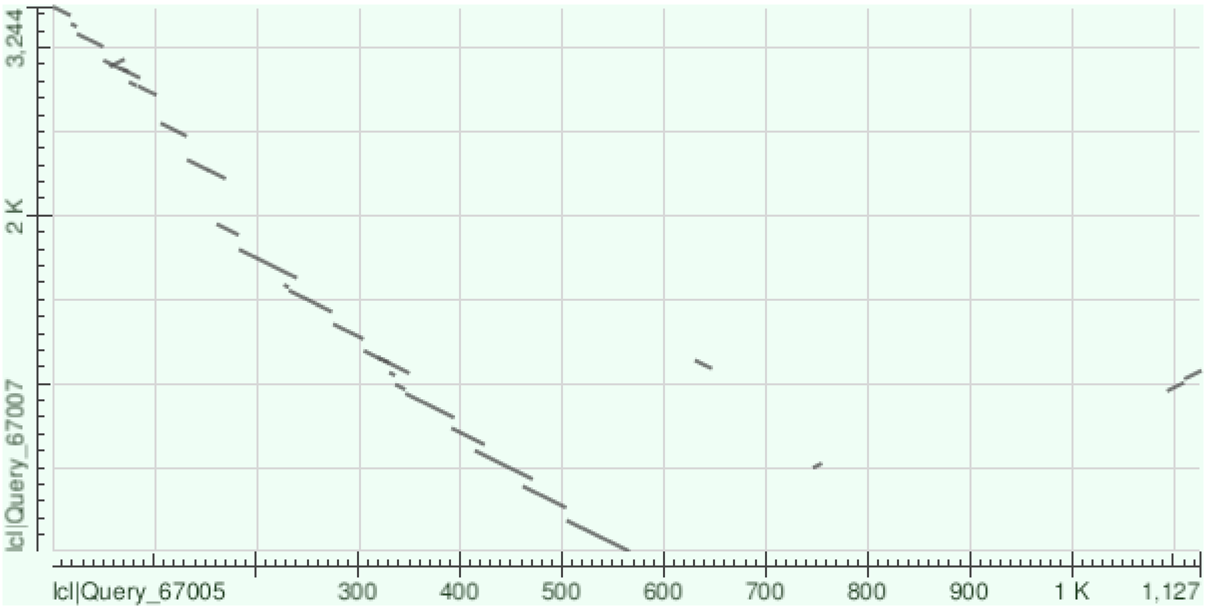
lcl|Query_67007
3,244
2 K
lcl|Query_67005
300
400
500
600
700
800
900
1 K
1,127

E

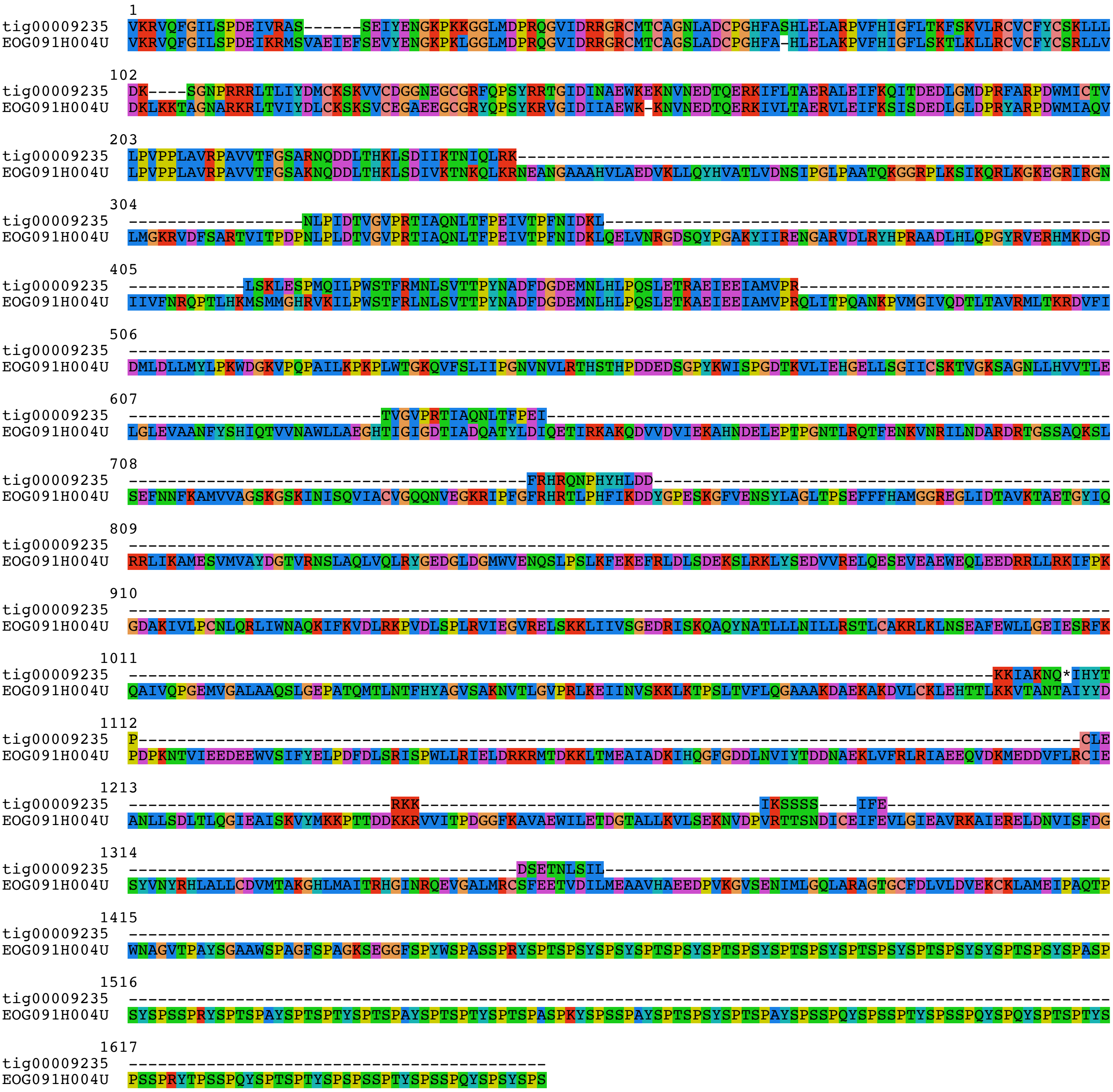
tig00009235
EOG091H004U
